# Retraction: Advanced Glycation End Products Induce Peroxisome Proliferator-Activated Receptor γ Down-Regulation-Related Inflammatory Signals in Human Chondrocytes via Toll-Like Receptor-4 and Receptor for Advanced Glycation End Products

**DOI:** 10.1371/journal.pone.0303122

**Published:** 2024-05-01

**Authors:** Ying Ju Chen, Meei Ling Sheu, Keh Sung Tsai, Rong Sen Yang, Shing Hwa Liu

Following the publication of this article [1], concerns were raised that some loading controls presented in Figs 1–5 and 7–10 appear similar, despite often representing different experimental conditions. Specifically,

- The β-actin panel in Fig 1D appears similar to the β-actin panel in Fig 8E.
- The α-tubulin panels in Figs 2B, 2C, 2D, 3A, 3B, 4B, 5A and 5D appear similar to each other.
- The α-tubulin panels in Figs 2A, 5B, 5C, and the β-actin panel in Fig 7B appear similar to each other.
- The Fig 3D β-actin panel and lanes 2–7 of the Fig 9C β-actin panel appear similar to each other.
- The β-actin panels in Fig 7C and 7D appear similar to each other.
- The β-actin panels in Fig 10B and 10C appear similar to each other.

In addition, the figure legends for Figs 1–10 state that protein expression was determined by densitometric analysis and that the results were corrected using α-tubulin and β-actin loading controls. Considering the concerns regarding similarity of loading control panels presented for the figures mentioned above, the validity and reliability of the change in fold of expression results presented in the respective Figs 1–10 are also in question.

Corresponding author Shing Hwa Liu explained that, due to author negligence, the same internal controls were used when preparing the figures for publication. They stated that the original data underlying the published results are no longer available, and provided data obtained from repeat experiments carried out at a later date. However, in the absence of the original underlying data, the *PLOS ONE* Editors consider that the repeat study data provided in this case constitute a new replicate study, while the concerns raised about the originally published data are not resolved.

In light of the unresolved concerns that question the validity and reliability of the published data, the authors retract this article.

All authors agreed with the retraction.
